# 4-(1*H*-Tetra­zol-5-yl)benzoic acid monohydrate

**DOI:** 10.1107/S1600536808019053

**Published:** 2008-06-28

**Authors:** Guo-Qing Li, A-Qing Wu, Yan Li, Fa-Kun Zheng, Guo-Cong Guo

**Affiliations:** a Chemistry and Life Sciences School, Quanzhou Normal University, Quanzhou, Fujian 362000, People’s Republic of China; b State Key Laboratory of Structural Chemistry, Fujian Institute of Research on the Structure of Matter, Chinese Academy of Sciences, Fuzhou, Fujian 350002, People’s Republic of China

## Abstract

The asymmetric unit of the title compound, C_8_H_6_N_4_O_2_·H_2_O, consists of one 4-(1*H*-tetra­zol-5-yl)benzoic acid mol­ecule and one water mol­ecule. Hydrogen-bonding and π–π stacking (centroid–centroid distance between tetra­zole and benzene rings = 3.78 Å) inter­actions link the mol­ecules into a three-dimensional network.

## Related literature

For general background, see: James *et al.* (2003 ▶); Kitagawa & Matsuda (2007 ▶); Maspoch *et al.* (2007 ▶); Pan *et al.* (2006 ▶); Li *et al.* (2007 ▶). For related tetra­zole ligands, see: Demko *et al.* (2001 ▶).
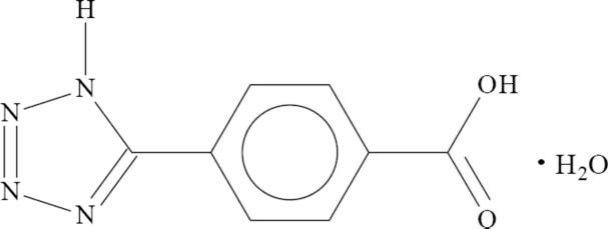


## Experimental

### 

#### Crystal data

C_8_H_6_N_4_O_2_·H_2_O
*M* *_r_* = 208.18
Monoclinic, 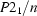

*a* = 4.914 (2) Å
*b* = 5.219 (2) Å
*c* = 34.720 (13) Å
β = 91.00 (3)°
*V* = 890.4 (6) Å^3^
*Z* = 4
Mo *K*α radiation
μ = 0.12 mm^−1^
*T* = 293 (2) K
0.20 × 0.10 × 0.10 mm

#### Data collection

Rigaku AFC-7R diffractometer
Absorption correction: ψ scan (*Psi* in **WinAFC Diffractometer Control Software**; Rigaku 2002 ▶) *T*_min_ = 0.927, *T*_max_ = 1.000 (expected range = 0.917–0.988)
3386 measured reflections
1576 independent reflections
1270 reflections with *I* > 2σ(*I*)
*R*_int_ = 0.028
3 standard reflections every 200 reflections intensity decay: 0.3%

#### Refinement

*R*[*F* ^2^ > 2σ(*F* ^2^)] = 0.037
*wR*(*F* ^2^) = 0.095
*S* = 1.01
1576 reflections
148 parameters
4 restraints
H atoms treated by a mixture of independent and constrained refinement
Δρ_max_ = 0.17 e Å^−3^
Δρ_min_ = −0.29 e Å^−3^

### 

Data collection: *WinAFC Diffractometer Control Software* (Rigaku, 2002 ▶); cell refinement: *WinAFC Diffractometer Control Software*; data reduction: *CrystalStructure* (Rigaku/MSC, 2004 ▶; program(s) used to solve structure: *SHELXTL* (Sheldrick, 2008 ▶); program(s) used to refine structure: *SHELXTL*; molecular graphics: *SHELXTL*; software used to prepare material for publication: *SHELXTL*.

## Supplementary Material

Crystal structure: contains datablocks global, I. DOI: 10.1107/S1600536808019053/sj2513sup1.cif

Structure factors: contains datablocks I. DOI: 10.1107/S1600536808019053/sj2513Isup2.hkl

Additional supplementary materials:  crystallographic information; 3D view; checkCIF report

## Figures and Tables

**Table 1:** Hydrogen-bond geometry (Å, °)

*D*—H⋯*A*	*D*—H	H⋯*A*	*D*⋯*A*	*D*—H⋯*A*
O1—H1⋯O2^i^	0.877 (10)	1.744 (10)	2.620 (2)	176 (3)
O1*W*—H1*WA*⋯N2^ii^	0.858 (10)	2.234 (16)	2.957 (2)	142 (2)
O1*W*—H1*WB*⋯N3^iii^	0.859 (10)	2.046 (10)	2.903 (2)	175 (2)

Symmetry codes: (i) 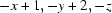; (ii) 
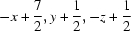
; (iii) 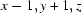.

**Fig. 1.**
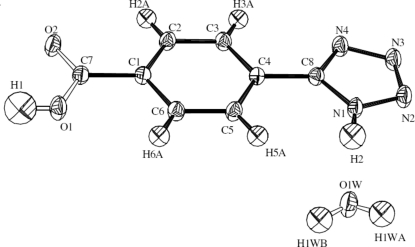
The molecular structure of I, with 30% probability displacement ellipsoids.

**Fig. 2.**
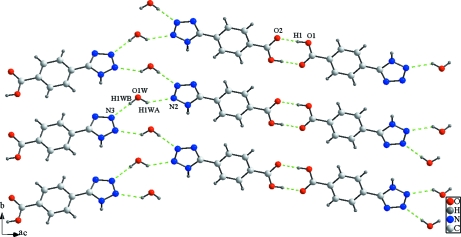
Packing of (I) into two-dimensional layers linked by O—H···N and O—H···O hydrogen bonds (green dashed lines).

**Fig. 3.**
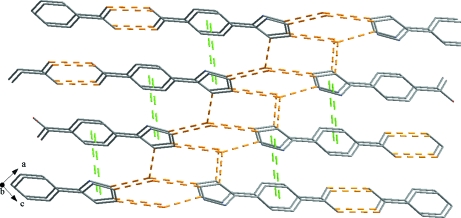
A three-dimensional framework for (I) linked by the π-π stacking interactions (green dashed lines). Hydrogen bonds are shown as yellow dashed lines.

## supplementary crystallographic information

### Comment

The current interest in crystal engineering of metal-organic coordination polymers (MOCPs) stems not only from their intriguing variety of architectures and topologies but also from their characteristic physical and/or chemical properties, including ferroelectricity, luminescence, magnetism, nonlinear optics, and gas storage, (James, *et al.* 2003; Kitagawa, *et al.* 2007; Maspoch, *et al.* 2007; Pan, *et al.* 2006; Li, *et al.* 2007). Multifunctional organic ligands are necessary for constructing such frameworks. Tetrazoles are versatile ligands due to their many potential donor atoms.  They can be synthesized easily by the reaction of a cyano group with NaN_3_ in the presence of ZnBr_2_ (Lewis acid) as a catalyst and water under reflux or hydrothermal reaction conditions (Demko, *et al.* 2001). Here, we report the synthesis and crystal structure of a new tetrazole [C_8_H_6_N_4_O_2_].H_2_O (I).

The asymmetric unit of  (I), consists of one crystallographically independent 4–5*H*-tetrazolyl-benzenecarboxylate molecule and one lattice water molecule (Figure 1). The molecular skeleton of I is essentially planar and the dihedral angle between the tetrazole and benzene rings is 0.16 °.  Two adjacent 4–5*H*-tetrazolyl-benzenecarboxylate molecules are linked to form a centrosymmetric dimer through O1—H1···O2  hydrogen bonds. These dimers are bridged by lattice water molecules through O1W—H1WA···N2 and O1W—H1WB···N3 hydrogen bonds to form a two-dimensional layer along the [0 1 0] and [7 0 1] directions, (Figure 2).  The layers are organized further by π-π stacking interactions between the tetrazole and benzene rings to form a three-dimensional framework. The two rings involved in the π-π stacking interactions are nearly parallel to each other, with a dihedral angle of 0.15 ° between them. The Cg1···Cg2^i^ distance is 3.78 Å where Cg1 and Cg2 are the centroids of the C1···C6 and C8/N1···N4 rings respectively (i = x-1, y, z).

### Experimental

A mixture of zinc bromide (225 mg, 1.0 mmol), Na(4-cba) (4-Hcba = 4-cyanobenzoic acid) (65 mg, 1.0 mmol) and NaN_3_ (65 mg, 1.0 mmol) in 10 ml water were transferred into a Teflon-line stainless steel autoclave and heated to 413 K for 3 days, then cooled to room temperature at the rate of 1 K/h. The resulting solid powder was acidified with HCl (2*M*) to give the target product.  Crystals were obtained by slow evaporation of the resulting solution.

### Refinement

The H atoms bound to O1W, O1 and N1 were located in a difference Fourier synthesis and refined with isotropic displacement parameters and the O(N)—H distances restrained to a target value of 0.86 (1) Å, and with *U*_iso_(H) of O1W being 1.2*U*_eq_(O1W). The remaining aromatic H atoms were positioned geometrically and refined using a riding model with d(C-H) = 0.93Å,  U_iso_=1.2U_eq_ (C).

### Crystal data

**Table:**

C_8_H_6_N_4_O_2_·H_2_O	*F*_000_ = 432
*M**_r_* = 208.18	*D*_x_ = 1.553 Mg m^−^^3^
Monoclinic, *P*2_1_/*n*	Mo *K*α radiation λ = 0.71073 Å
Hall symbol: -P 2yn	Cell parameters from 20 reflections
*a* = 4.914 (2) Å	θ = 12–30º
*b* = 5.219 (2) Å	µ = 0.12 mm^−^^1^
*c* = 34.720 (13) Å	*T* = 293 (2) K
β = 91.00 (3)º	Block, colorless
*V* = 890.4 (6) Å^3^	0.20 × 0.10 × 0.10 mm
*Z* = 4	

### Data collection

**Table:**

Rigaku AFC-7R diffractometer	*R*_int_ = 0.028
Radiation source: rotating-anode generator	θ_max_ = 25.0º
Monochromator: graphite	θ_min_ = 3.5º
*T* = 293(2) K	*h* = −1→5
ω–2θ scans	*k* = −6→6
Absorption correction: ψ scan(Psi in *WinAFC Diffractometer Control Software*; Rigaku 2002)	*l* = −41→41
*T*_min_ = 0.928, *T*_max_ = 1.000	3 standard reflections
3386 measured reflections	every 200 reflections
1576 independent reflections	intensity decay: 0.3%
1270 reflections with *I* > 2σ(*I*)	

### Refinement

**Table:**

Refinement on *F*^2^	Secondary atom site location: difference Fourier map
Least-squares matrix: full	Hydrogen site location: inferred from neighbouring sites
*R*[*F*^2^ > 2σ(*F*^2^)] = 0.038	H atoms treated by a mixture of independent and constrained refinement
*wR*(*F*^2^) = 0.095	*w* = 1/[σ^2^(*F*_o_^2^) + (0.0403*P*)^2^ + 0.366*P*] where *P* = (*F*_o_^2^ + 2*F*_c_^2^)/3
*S* = 1.01	(Δ/σ)_max_ < 0.001
1576 reflections	Δρ_max_ = 0.17 e Å^−^^3^
148 parameters	Δρ_min_ = −0.29 e Å^−^^3^
4 restraints	Extinction correction: none
Primary atom site location: structure-invariant direct methods	

### Special details

**Table:**

Geometry. All e.s.d.'s (except the e.s.d. in the dihedral angle between two l.s. planes) are estimated using the full covariance matrix. The cell e.s.d.'s are taken into account individually in the estimation of e.s.d.'s in distances, angles and torsion angles; correlations between e.s.d.'s in cell parameters are only used when they are defined by crystal symmetry. An approximate (isotropic) treatment of cell e.s.d.'s is used for estimating e.s.d.'s involving l.s. planes.
Refinement. Refinement of *F*^2^ against ALL reflections. The weighted *R*-factor *wR* and goodness of fit *S* are based on *F*^2^, conventional *R*-factors *R* are based on *F*, with *F* set to zero for negative *F*^2^. The threshold expression of *F*^2^ > σ(*F*^2^) is used only for calculating *R*-factors(gt) *etc*. and is not relevant to the choice of reflections for refinement. *R*-factors based on *F*^2^ are statistically about twice as large as those based on *F*, and *R*- factors based on ALL data will be even larger.

### Fractional atomic coordinates and isotropic or equivalent isotropic displacement parameters (Å^2^)

**Table:**

	*x*	*y*	*z*	*U*_iso_*/*U*_eq_	
O1W	1.3075 (3)	1.2115 (3)	0.22404 (4)	0.0510 (4)	
H1WA	1.400 (4)	1.292 (4)	0.2414 (5)	0.061*	
H1WB	1.175 (3)	1.304 (4)	0.2153 (6)	0.061*	
O1	0.6331 (3)	1.1354 (3)	0.04240 (4)	0.0456 (4)	
H1	0.509 (4)	1.157 (6)	0.0242 (6)	0.100*	
O2	0.7482 (3)	0.7840 (3)	0.00986 (3)	0.0453 (4)	
N1	1.6033 (3)	0.8366 (3)	0.18939 (4)	0.0363 (4)	
H2	1.514 (4)	0.965 (3)	0.1990 (6)	0.068*	
N2	1.8032 (3)	0.7255 (3)	0.21010 (4)	0.0429 (4)	
N3	1.8788 (3)	0.5272 (3)	0.19041 (4)	0.0439 (4)	
N4	1.7318 (3)	0.5062 (3)	0.15713 (4)	0.0396 (4)	
C1	0.9801 (3)	0.8752 (3)	0.06869 (4)	0.0301 (4)	
C2	1.1494 (4)	0.6629 (3)	0.06537 (5)	0.0345 (4)	
H2A	1.1350	0.5576	0.0438	0.041*	
C3	1.3393 (4)	0.6083 (3)	0.09416 (5)	0.0338 (4)	
H3A	1.4531	0.4669	0.0918	0.041*	
C4	1.3602 (3)	0.7647 (3)	0.12651 (4)	0.0289 (4)	
C5	1.1923 (4)	0.9778 (3)	0.12961 (5)	0.0340 (4)	
H5A	1.2076	1.0839	0.1511	0.041*	
C6	1.0034 (3)	1.0320 (3)	0.10098 (5)	0.0332 (4)	
H6A	0.8908	1.1742	0.1033	0.040*	
C7	0.7755 (3)	0.9317 (3)	0.03801 (5)	0.0319 (4)	
C8	1.5608 (3)	0.7022 (3)	0.15704 (4)	0.0297 (4)	

### Atomic displacement parameters (Å^2^)

**Table:**

	*U*^11^	*U*^22^	*U*^33^	*U*^12^	*U*^13^	*U*^23^
O1W	0.0586 (9)	0.0478 (9)	0.0459 (8)	0.0216 (7)	−0.0217 (6)	−0.0168 (6)
O1	0.0509 (8)	0.0439 (8)	0.0414 (7)	0.0187 (7)	−0.0173 (6)	−0.0079 (6)
O2	0.0510 (8)	0.0496 (8)	0.0348 (7)	0.0147 (7)	−0.0156 (6)	−0.0130 (6)
N1	0.0399 (9)	0.0372 (9)	0.0313 (8)	0.0110 (7)	−0.0114 (6)	−0.0040 (6)
N2	0.0458 (9)	0.0444 (9)	0.0380 (8)	0.0125 (8)	−0.0152 (7)	−0.0023 (7)
N3	0.0463 (9)	0.0443 (9)	0.0407 (8)	0.0143 (8)	−0.0138 (7)	−0.0013 (7)
N4	0.0430 (9)	0.0381 (9)	0.0374 (8)	0.0117 (7)	−0.0102 (7)	−0.0024 (7)
C1	0.0313 (9)	0.0303 (9)	0.0286 (8)	0.0010 (7)	−0.0027 (7)	−0.0002 (7)
C2	0.0396 (10)	0.0334 (10)	0.0302 (9)	0.0043 (8)	−0.0055 (7)	−0.0071 (7)
C3	0.0350 (9)	0.0318 (9)	0.0346 (9)	0.0077 (8)	−0.0052 (7)	−0.0029 (7)
C4	0.0289 (8)	0.0300 (9)	0.0277 (8)	0.0008 (7)	−0.0035 (7)	0.0016 (7)
C5	0.0399 (10)	0.0323 (9)	0.0294 (9)	0.0050 (8)	−0.0070 (7)	−0.0064 (7)
C6	0.0360 (9)	0.0297 (9)	0.0337 (9)	0.0080 (8)	−0.0052 (7)	−0.0027 (7)
C7	0.0333 (9)	0.0326 (9)	0.0296 (9)	0.0035 (8)	−0.0031 (7)	−0.0011 (7)
C8	0.0322 (9)	0.0285 (9)	0.0284 (8)	0.0020 (8)	−0.0029 (7)	0.0010 (7)

### Geometric parameters (Å, °)

**Table:**

O1W—H1WA	0.858 (10)	C1—C2	1.392 (2)
O1W—H1WB	0.859 (10)	C1—C7	1.482 (2)
O1—C7	1.283 (2)	C2—C3	1.385 (2)
O1—H1	0.877 (10)	C2—H2A	0.9300
O2—C7	1.250 (2)	C3—C4	1.391 (2)
N1—C8	1.337 (2)	C3—H3A	0.9300
N1—N2	1.339 (2)	C4—C5	1.390 (2)
N1—H2	0.872 (10)	C4—C8	1.471 (2)
N2—N3	1.298 (2)	C5—C6	1.378 (2)
N3—N4	1.356 (2)	C5—H5A	0.9300
N4—C8	1.324 (2)	C6—H6A	0.9300
C1—C6	1.391 (2)		
			
H1WA—O1W—H1WB	111 (2)	C4—C3—H3A	120.0
C7—O1—H1	113 (2)	C5—C4—C3	119.78 (15)
C8—N1—N2	109.02 (14)	C5—C4—C8	120.84 (15)
C8—N1—H2	130.9 (16)	C3—C4—C8	119.38 (15)
N2—N1—H2	119.7 (15)	C6—C5—C4	120.14 (15)
N3—N2—N1	106.08 (14)	C6—C5—H5A	119.9
N2—N3—N4	111.08 (14)	C4—C5—H5A	119.9
C8—N4—N3	105.55 (14)	C5—C6—C1	120.35 (16)
C6—C1—C2	119.63 (15)	C5—C6—H6A	119.8
C6—C1—C7	120.49 (15)	C1—C6—H6A	119.8
C2—C1—C7	119.88 (15)	O2—C7—O1	123.55 (15)
C3—C2—C1	120.02 (16)	O2—C7—C1	120.08 (15)
C3—C2—H2A	120.0	O1—C7—C1	116.37 (14)
C1—C2—H2A	120.0	N4—C8—N1	108.27 (14)
C2—C3—C4	120.08 (16)	N4—C8—C4	126.17 (15)
C2—C3—H3A	120.0	N1—C8—C4	125.55 (15)

### Hydrogen-bond geometry (Å, °)

**Table:**

*D*—H···*A*	*D*—H	H···*A*	*D*···*A*	*D*—H···*A*
O1—H1···O2^i^	0.877 (10)	1.744 (10)	2.620 (2)	176 (3)
O1W—H1WA···N2^ii^	0.858 (10)	2.234 (16)	2.957 (2)	142 (2)
O1W—H1WB···N3^iii^	0.859 (10)	2.046 (10)	2.903 (2)	175 (2)

Symmetry codes: (i) −*x*+1, −*y*+2, −*z*; (ii) −*x*+7/2, *y*+1/2, −*z*+1/2; (iii) *x*−1, *y*+1, *z*.
